# Ratings of perceived exertion (RPE) from a submaximal 20-m shuttle-run test accurately predict children’s VO_2peak_, but when should we stop the test?

**DOI:** 10.1007/s00421-024-05618-9

**Published:** 2024-09-30

**Authors:** Daiki Kasai, Margarita D. Tsiros, Roger Eston, Gaynor Parfitt

**Affiliations:** 1 Alliance for Research in Exercise, Nutrition and Activity (ARENA), University of South Australia, City East Campus, Cnr of North Terrace & Frome Rd, Adelaide, SA 5001 Australia; 2 Innovation, Implementation and Clinical Translation in Health (IIMPACT), University of South Australia, City East Campus, Cnr of North Terrace & Frome Rd, Adelaide, SA 5001 Australia

**Keywords:** RPE, Affect, Oxygen uptake, Fitness test

## Abstract

**Purpose:**

The purpose of the study was to explore the validity, test–retest reliability and affective responses of a submaximal 20-m shuttle-run test (20mSRT) stopped at 6 on the Eston–Parfitt (EP) scale. The secondary aim was to examine and compare two submaximal 20mSRT protocols with different RPE end points (EP6 vs. EP7) using previously published data.

**Methods:**

Twenty-five children (16 boys; 13.4 ± 1.0 years; 162.1 ± 8.7 cm; 49.1 ± 6.6 kg) completed three exercise tests (graded exercise test [GXT], 2 submaximal 20mSRT). The EP scale and Feeling scale were used to measure RPE and affect, respectively. The two submaximal 20mSRTs were stopped after participants reported EP6. Individual speed–RPE relationships from the submaximal 20mSRTs were linearly regressed to predict peak speed and then used to estimate VO_2peak_. Previously published data (*n* = 25) used comparable methods, except that the participants stopped at EP7.

**Results:**

In the EP6 protocol, a two-factor repeated measures ANOVA revealed non-significant Test and Sex main effects (*p* > 0.05). Reliability analysis revealed intraclass correlation coefficient of ~ 0.7 (95%CI [0.432,0.867], *p* < 0.001) between the submaximal 20mSRTs. Significant differences in end-test affect between the GXT and submaximal 20mSRTs were found (*p* < 0.001), with GXT more negative. ANOVA revealed no significant differences in end-test affect between EP6 and EP7 protocols; however, frequency count analysis revealed EP6 to result in more positive end-test affect.

**Conclusion:**

Submaximal 20mSRT utilising RPE may offer valid predictions in VO_2peak_ while minimising negative affect. Test end points of EP6 and EP7 both offer valid predictions in VO_2peak_. EP6 may be more beneficial in avoiding negative affect, even though a reduction in test–retest reliability was observed.

## Introduction

Cardiorespiratory fitness is an important indicator of health (Raghuveer et al. 2020). In childhood, cardiorespiratory fitness is particularly important for optimal development and is inversely associated with psychological well-being and cognitive function (Chen et al. 2022), while also being a predictor of future health outcomes (Hőgstrőm et al. 2016).

Direct measurement of peak oxygen uptake (VO_2peak_), through a laboratory-based graded exercise test (GXT), is considered the gold standard assessment of cardiorespiratory fitness in children (Noonan and Dean 2000). However, laboratory-based exercise testing requires expensive equipment and expertise and is therefore not portable nor suited for mass testing (Tomkinson and Olds 2008). Field-based fitness tests are a pragmatic solution to laboratory-based testing methods and rely on measures such as heart rate (HR) and performance metrics (i.e. distance, speed) to predict VO_2peak_ through standardised equations. Field-based fitness testing is commonly utilised in physical education curriculums internationally (Cale et al. 2014). The 20-m ‘shuttle-run’ test (20mSRT) is one of the most widely used field-based tests to assess cardiorespiratory fitness (Lang et al. 2018). However, the 20mSRT is a maximal exercise test that requires participants to exercise until volitional exhaustion which inherently induces unpleasant emotional responses in children (Kasai et al. 2023).

The subjective experience of pleasure/displeasure (i.e. affective valence) that an individual feels during exercise is important to consider (Ekkekakis and Petruzzello 2002; Ekkekakis et al. 2020), especially as the acute affective response during exercise may impact future exercise behaviour (Ekkekakis et al. 2011; Rhodes and Kates 2015). In both adults and children, a 1-unit increase in positive affective response during an exercise bout was associated with ~ 30 min of additional MVPA per week (Williams et al. 2008; Schneider et al. 2009).

During exercise, affective response is the result of the interaction between our cognitive processes and interoceptive cues (e.g. incoming signals from various somatosensory receptors) that arise depending on the intensity of the exercise bout (Ekkekakis 2003). According to the dual-mode theory (Ekkekakis 2003), there are three distinct domains of exercise intensity (i.e. moderate, heavy and severe) that are characterised by the underlying metabolic processes (Ekkekakis et al. 2020). The ‘moderate’ domain of exercise includes intensities up to and below the ventilatory threshold (VT). Exercise within the moderate domain is likely to produce positive affective responses (Ekkekakis 2003). The second domain (‘heavy’) is characterised as intensities between the VT and respiratory compensation point. Affective responses in the heavy domain are subject to high variability due to interindividual differences in our cognitive processes and how the intensified interoceptive cues are interpreted (Tempest and Parfitt 2017). The final domain (‘severe’) of exercise intensity extends beyond the respiratory compensation point where affective responses are uniformly negative due to the interoceptive cues overriding cognitive processes in an attempt to avoid physiological harm (Ekkekakis et al. 2005). The relationship between exercise that is at or beyond the ‘severe’ domain and negative affect is important to consider, as children may be exposed to these intensities frequently through standardised maximal fitness testing practices in schools.

Whilst the subjective experience of ‘how the exercise feels’ is important for promoting exercise adherence, our ability to discern ‘how hard we are working during the exercise’ has significant utility. Ratings of perceived exertion (RPE) is the ability to detect and interpret sensations that arise from the body during exercise to evaluate how difficult the bout of exercise feels at any given point in time (Eston and Parfitt 2018). In children, RPE have been used to estimate cardiorespiratory fitness/exercise performance, monitor internal training loads, and control exercise intensity (Kasai et al. 2021). The utility of RPE is founded on its strong relationship with objective markers of exercise (e.g. VO_2_, HR, work rate). In regard to predicting cardiorespiratory fitness (i.e. VO_2peak_), the relationship between RPE and VO_2_ can be extrapolated to a theoretical end point. The efficacy of predicting VO_2max/peak_ during laboratory-based exercise has been examined in adults (Coquart et al. 2014) and children (Lambrick et al. 2016). Our recently published work (Kasai et al. 2023) found that a submaximal 20mSRT, terminated upon participants reporting EP7 (“Very hard”) on the ‘Eston–Parfitt 0–10 RPE’ scale (Eston and Parfitt 2007), predicted VO_2peak_ in children accurately. The submaximal 20mSRT protocol also had excellent reliability (ICC ~ 0.9). Importantly, mean end-test affective responses observed from the submaximal 20mSRTs were less negative in comparison to the traditional, maximal 20mSRT. However, the mean end-test affective responses from the submaximal 20mSRTs were still in the negative domain, particularly for girls (Kasai et al. 2023).

As even a 1-unit change in affective response during exercise may have a significant impact on future exercise behaviour (Williams et al. 2008; Schneider et al. 2009), a submaximal 20mSRT terminated at a lower perceptual end point (i.e. lower exercise intensity) may be of benefit as it may be less ‘affectually aversive’ according to the dual-mode theory. However, while it has not been empirically explored, previous research suggests that a lower test end point may lead to less accurate and less reliable predictions of VO_2peak_ (Faulkner et al. 2007; Lambrick et al. 2016). Predictions of VO_2peak_ are generally more accurate at higher exercise intensities due to a stronger relationship between RPE and physiological markers of exercise (e.g. HR, VO_2_) (Eston and Williams 1988). In this regard, in the treadmill study by Lambrick et al. (2016), predictions of VO_2peak_ extrapolated from EP5 (~ 80% VO_2peak_) were less accurate than EP7. In adults, Faulkner et al. (2007) found reductions in ICC for lower perceptual ranges. Nevertheless, if ending a submaximal fitness test at a lower perceptual end point (EP6) is acceptably accurate and reliable and results in less aversive affective responses, it could provide an alternative to maximal fitness testing for children.

This study aimed to examine the validity, test–retest reliability, and affective responses of a submaximal 20mSRT protocol that was terminated at EP6. Our secondary aim was to examine data from our recently published work (Kasai et al. 2023) and compare both 20mSRT protocols with different perceptual end points. We hypothesised that the submaximal 20mSRT protocol stopped at EP6 would accurately and reliably predict VO_2peak_ in children aged 12–14 years, but would be less accurate compared to the EP7 protocol. However, we also hypothesised that the end-test affective responses in the EP6 protocol would be less affectually aversive in comparison to a maximal exercise test (i.e. GXT) and the EP7 protocol.

## Methods

### Participants

Participants (32 healthy boys and girls aged 12–14 y, with no known illnesses, pre-existing injuries, or disabilities) were recruited through advertisements on social media platforms (including sponsored advertisements) from October 2022 to September 2023. Parents of potential participants were sent information sheets and a screening questionnaire to deem eligibility. The study received ethics approval from the University of South Australia Human Research Ethics Committee (Ethics no. 204723) and was conducted in accordance with the Declaration of Helsinki. Written consent and assent were obtained from the parent/legal guardian and participants, respectively.

### Procedures

Participants completed three exercise trials in a non-randomised order: a graded exercise test (GXT), followed by two submaximal modified 20mSRTs. Each trial was separated by a minimum of 48 h (median = 12 days [IQR, 7–22 days]). The ordering of the exercise trials was kept consistent between participants (i.e. non-randomised) to allow ‘perceptual anchoring’ and to give participants an opportunity to physically experience and familiarise themselves with the range of sensations that correspond to a given RPE level (Eston and Lamb 2000). Height and mass were recorded using a stadiometer (SECA 213, Ecomed, NSW, Australia) and scales (BC-148, Tanita, Tokyo, Japan) during the first visit.

#### Graded exercise test

On the first visit to the laboratory, a GXT to volitional exhaustion was conducted on a motorised treadmill (PPS Med 55, Woodway USA Inc., WI, USA) to assess peak oxygen uptake (VO_2peak_). The attainment criteria for a maximal effort during the GXT were: a respiratory exchange ratio (RER) ≥ 1.0, reported RPE at 9 or 10, and  ≥ 90% of age-predicted maximal HR based on Tanaka et al. (2001) prediction equation (HR_max_ = 208 − 0.7 * age) (Armstrong and Fawkner 2007). Before commencing the GXT, participants were given demonstrations of safety procedures and familiarised with the treadmill. The GXT commenced at 4 km/h and increased by 1 km/h every minute up to 8 km/h, whereafter the speed increased by 0.5 km/h (Kasai et al. 2023). The treadmill gradient was kept constant at 1% to replicate the energy cost of overground running (Jones and Doust 1996). Breath-by-breath respiratory gas data were collected (Metalyzer 3B; Cortex Biophysik GmbH, Leipzig, Germany) with a paediatric face mask (Hans Rudolph Inc., Kansas, USA). Participants reported their RPE and affect using the Eston–Parfitt RPE scale and Feeling scale, respectively, during the last 15 s of each minute.

#### Modified 20-m shuttle-run test

On the second and third visit, participants performed the modified 20mSRT protocol individually to ascertain test–retest reliability. Prior to starting each trial, participants were informed that the test would stop when they reported a 6 on the E–P scale. The modified 20mSRT protocol started at 4 km/h and increased by 1 km/h every minute up to 8 km/h, whereafter the speed increased by 0.5 km/h every minute. Participants reported their RPE and affect prior to starting each trial and during the last levels of each stage prior to beginning the next speed, until they reported a 6 on the E–P scale, when the test was stopped.

Data from this study were compared with the study of Kasai et al. (2023) involving 25 children (*n* = 14 boys, *n* = 11) aged 12–14 years, which followed the same  exact methodology, with the exception of the submaximal end point of EP7.

### Material

#### Eston–Parfitt RPE scale

The Eston–Parfitt (E–P) RPE scale was used to measure perceived exertion during all three trials. The E–P scale is a pictorial 0–10 scale that shows characters at several stages of exertion along a progressively increasing slope with verbal anchors ranging from 0 (“very, very easy”) to 10 (“so hard I am going to stop”). The E–P scale has previously demonstrated good reliability in the production of exercise intensity (ICC = 0.71–0.76) (Eston and Parfitt 2007) and has been validated for quantifying overall perceived exertion in children during treadmill exercise (*R*^*2*^ = 0.96) (Lambrick et al. 2011) and overground running (Lambrick et al. 2013).The E–P scale has previously been used to predict VO_2peak_ in children during a laboratory-based graded exercise test (Lambrick et al. 2016) and in our recently published work that utilised the E–P scale during a 20mSRT that was terminated at EP7 (Kasai et al. 2023).

#### Feeling scale

Affective valence during each trial was recorded using the ‘Feeling scale’ (FS) (Hardy and Rejeski 1989). The FS is an 11-point numerical scale ranging from − 5 (very bad) and 0 (neutral) to + 5 (very good). The FS has been successfully used in studies with children to assess affective valence (Sheppard and Parfitt 2008; Schneider et al. 2009; Schneider and Graham 2009; Benjamin et al. 2012; Kasai et al. 2023).

Participants were introduced and re-familiarised on how to employ both the E–P scale and the FS prior to starting each session.

### Data analysis

Breath-by-breath data from the GXT were collapsed into 10-s bins. VO_2peak_ was determined as the highest 10-s value observed. Independent samples *t* tests were conducted using SPSS (ver.28, IBM Analytics, USA) to examine sex differences on descriptive physiological data. A significance level of 0.05 was set for all statistical analyses. Where sphericity was violated, the Greenhouse–Geisser correction factor was applied. Repeated measures planned comparisons were conducted to identify main effects or interactions.

#### Validity of VO_*2peak*_ predictions from submaximal 20mSRT

To examine the validity of predicting VO_2peak_ using RPE reported during the submaximal 20mSRT, a linear regression analysis was performed using Microsoft Excel ver. 2308 (Microsoft Corp, Redmond, USA). RPE and the corresponding speed after 8 km/h during the 20mSRT were linearly regressed to obtain the constant and *b*-coefficient. The linear regression was then extrapolated to EP9 and 10 to predict peak speed. Peak speed values at EP9 and 10 were then used to estimate VO_2peak_ using ACSM’s metabolic equation (ACSM 2007). Extrapolation end points of EP9 and 10 were used as children typically report terminal RPE values that are lower than the absolute maximal values following termination of a maximal bout of exercise (Lambrick et al. 2011). Measured VO_2peak_ from the GXT and estimated VO_2peak_ at both EP9 and EP10 from the first modified 20mSRT trial were analysed via a two-factor repeated measures analysis of variance (ANOVA) [Sex(2) x Test(3)] using SPSS.

#### Test–retest reliability of the submaximal 20mSRT

The test–retest reliability between the two submaximal 20mSRT trials were examined by calculating a two-way mixed effects intraclass correlation coefficient (ICC) based on a single measurement, on predicted peak speeds at EP9 and EP10 via SPSS. ICC estimates were used to interpret the results. Specifically, values less than 0.5 indicate poor reliability, 0.5–0.75 indicate moderate reliability, 0.75–0.9 indicate good reliability, and values greater than 0.9 indicate excellent reliability (Koo and Li 2016).

#### Affective responses analysis

##### Submaximal 20mSRTs at EP6 vs. GXT

To assess the difference in affective responses during each trial, a three-factor mixed-model ANOVA [Sex (2) × Time (2) × Trial (3)], with repeated measures on Time and Trial was conducted.

##### Comparison between two submaximal 20mSRT protocols

A two-factor MANOVA [Sex (2) × Protocol (2)] on descriptive physiological data was conducted to analyse the difference between the two sample groups at EP6 (current study) and EP7 (Kasai et al. 2023). To examine the difference in end-test affective responses between the two submaximal 20mSRT protocols that were stopped at EP6 (current study) and EP7 (Kasai et al. 2023), a four-factor mixed model ANOVA [Protocol (2) × Sex (2) × Trial (2) × Time], with repeated measures on Trial was conducted.

Finally, the interindividual variability in affective responses between the two submaximal 20mSRT protocols were assessed by analysing the range of negativity in FS scores reported and a frequency count of the number of individuals who reported negative affective responses.

## Results

From the 32 enrolled participants, 3 dropped out and 4 were excluded from the analysis due to equipment failure or not completing the protocol as intended. Therefore, 25 children (*n* = 16 boys) were included in the analysis. Independent samples *t* test revealed no significant differences between boys and girls (Table 1) for any descriptive variables.

**Table 1:** Descriptive characteristics of participants (mean ± SD)

Variable	All (n = 25)	Boys (*n* = 16)	Girls (*n* = 9)
Age (y)	13.4 ± 1.0	13.5 ± 1.0	13.2 ± 0.9
Height (cm)	162.1 ± 8.7	164.5 ± 8.5	157.9 ± **7**.8
Mass (kg)	49.1 ± 6.6	50.7 ± 6.3	46.1 ± 6.4
Body mass Index (BMI; kg·m^−2^)	18.6 ± 1.8	18.7 ± 1.9	18.4 ± 1.6
VO_2peak_ (mL·kg^−1^·min^−1^)	45.8 ± 5.4	47.1 ± 5.9	43.3 ± 3.4
HR_max_ (beats.min^−1^)	192.3 ± 7.9	192.6 ± 8.1	191.7 ± 8.1
RER	1.10 ± 0.06	1.11 ± 0.05	1.09 ± 0.06
VT (mL·kg^−1^·min^−1^)	30.3 ± 3.5	31.2 ± 3.8	28.6 ± 2.2
VT as % of VO_2peak_ (%)	66.2 ± 3.1	66.3 ± 3.2	66.1 ± 3.0
RPE at VT (GXT)	3.9 ± 0.8	3.9 ± 0.9	3.8 ± 0.6
Terminal RPE (GXT)	9.3 ± 0.7	9.4 ± 0.6	9.1 ± 0.8
Peak GXT speed (km·h^−1^)	12.1 ± 1.5	12.4 ± 1.4	11.6 ± 1.4

Note: no significant differences (*p* < 0.05) between boys and girls

*VO*_*2peak*_ peak oxygen uptake, *HR*_*max*_ maximal heart rate, *RER* respiratory exchange ratio, *VT* ventilatory threshold, *RPE* ratings of perceived exertion, *GXT* graded exercise test

### Prediction of VO_***2peak***_ at EP6

The two-factor repeated measures ANOVA demonstrated non-significant Test (*F*_1.016, 23.375_ = 2.922, *p* > 0.05, η_p_^2^ = 0.113) and Sex main effects (*F*_1, 23_ = 1.515, *p* > 0.05, η_p_^2^ = 0.062). Test by Sex interactions were also not significant (*F*_1.016, 23.375_ = 1.299, *p* > 0.05, η_p_^2^ = 0.053). Mean differences between measured VO_2_peak during the GXT and predicted VO_2_peak were 0.8 mL.kg.min and − 1.4 mL.kg.min at EP9 and EP10, respectively.

### Test–retest reliability of the submaximal 20mSRT at EP6

The ICC for predicted VO_2peak_ between the two submaximal 20mSRTs was 0.722 (95%CI; 0.463, 0.867) and 0.703 (95%CI; 0.432, 0.857) at EP9 and EP10, respectively (*p* < 0.001).

### Affective response analysis of submaximal 20mSRT at EP6

**Fig. 1:**
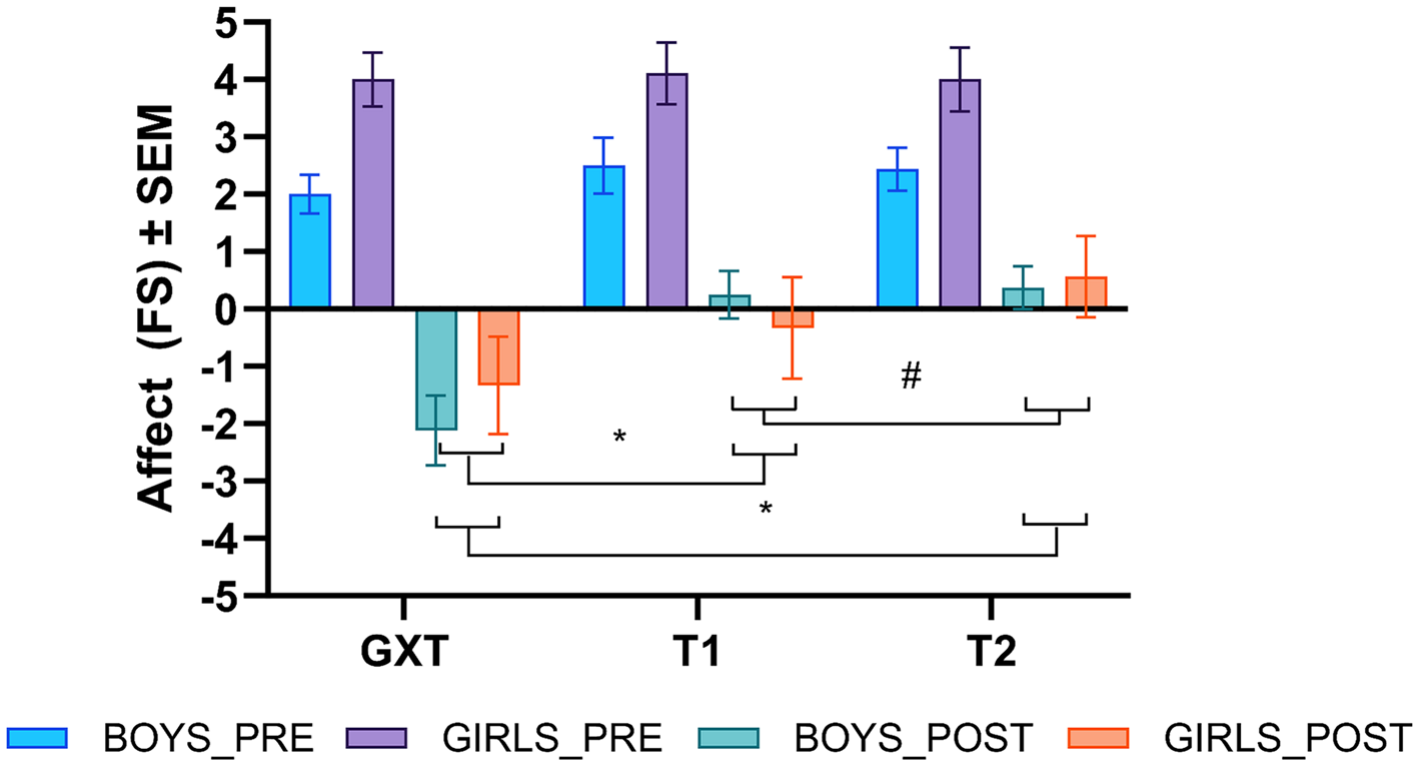
Pre-test and end-test affective responses (mean ± SE) of boys (*n* = 16) and girls (*n* = 9) performing the GXT and two submaximal 20mSRTs stopped at EP6.  =  depicts significant differences in the mean values between the two trials, * = significant differences (*p* < 0.001), # = significant differences (*p* < 0.01) Note. GXT: graded exercise test, T1: first trial of submaximal 20-m shuttle-run test, T2: second trial of submaximal 20-m shuttle-run test

The three-factor mixed model ANOVA revealed significant Trial (*F*_2, 46_ = 25.050, *p* < 0.001, η_p_^2^ = 0.521) and Time (*F*_1, 23_ = 63.195, *p* < 0.001, η_p_^2^ = 0.733) main effects. Time by Trial (*F*_1.586, 36.473_ = 18.061, *p* < 0.001, η_p_^2^ = 0.440) was also significant. All other interactions were non-significant (*p* > 0.05). Post hoc analyses revealed no significant differences in pre-test affective responses across all three trials (*p* > 0.05). However, there were significant differences in end-test affective responses between the GXT and the 1st (mean difference = − 1.69 FS units, *p* < 0.001) and 2nd (mean difference = − 2.19 FS units, *p* < 0.001) submaximal 20mSRT trials. Significant differences in end-test affective responses were also found between the two submaximal 20mSRTs (mean difference = − 0.51 FS units, *p* = 0.009) (Fig. 1).

### Comparison between the EP6 and EP7 submaximal 20mSRT protocols

The two-factor MANOVA revealed significant Sex (*F*_8, 39_ = 2.397, *p* = 0.033, η_p_^2^ = 0.330) and Protocol (*F*_8, 39_ = 2.355, *p* = 0.036, η_p_^2^ = 0.326) main effects. Sex by Protocol interactions were not significant (*p* > 0.05). Post-hoc analyses show that the EP7 group were younger (mean difference = − 0.54 years, *p* = 0.03) and boys in the EP7 group had a higher measured VO_2peak_ compared to the girls (mean difference = 7.151 mL.kg.min, *p* < 0.001). No other significant differences were found.

The four-factor mixed model ANOVA to compare end-test affective responses obtained from the current study and our previously published study (Kasai et al. 2023) protocols showed no significant main effects or interactions. However, the Trial (*F*_1.0, 46.0_ = 3.519, *p* = 0.067, η_p_^2^ = 0.071) main effect, Trial by Protocol (*F*_1.0, 46.0_ = 3.800, *p* = 0.057, η_p_^2^ = 0.076) and Trial by Sex by Protocol (*F*_1.0, 46.0_ = 3.053, *p* = 0.087, η_p_^2^ = 0.062) interactions approached significance.

**Fig. 2:**
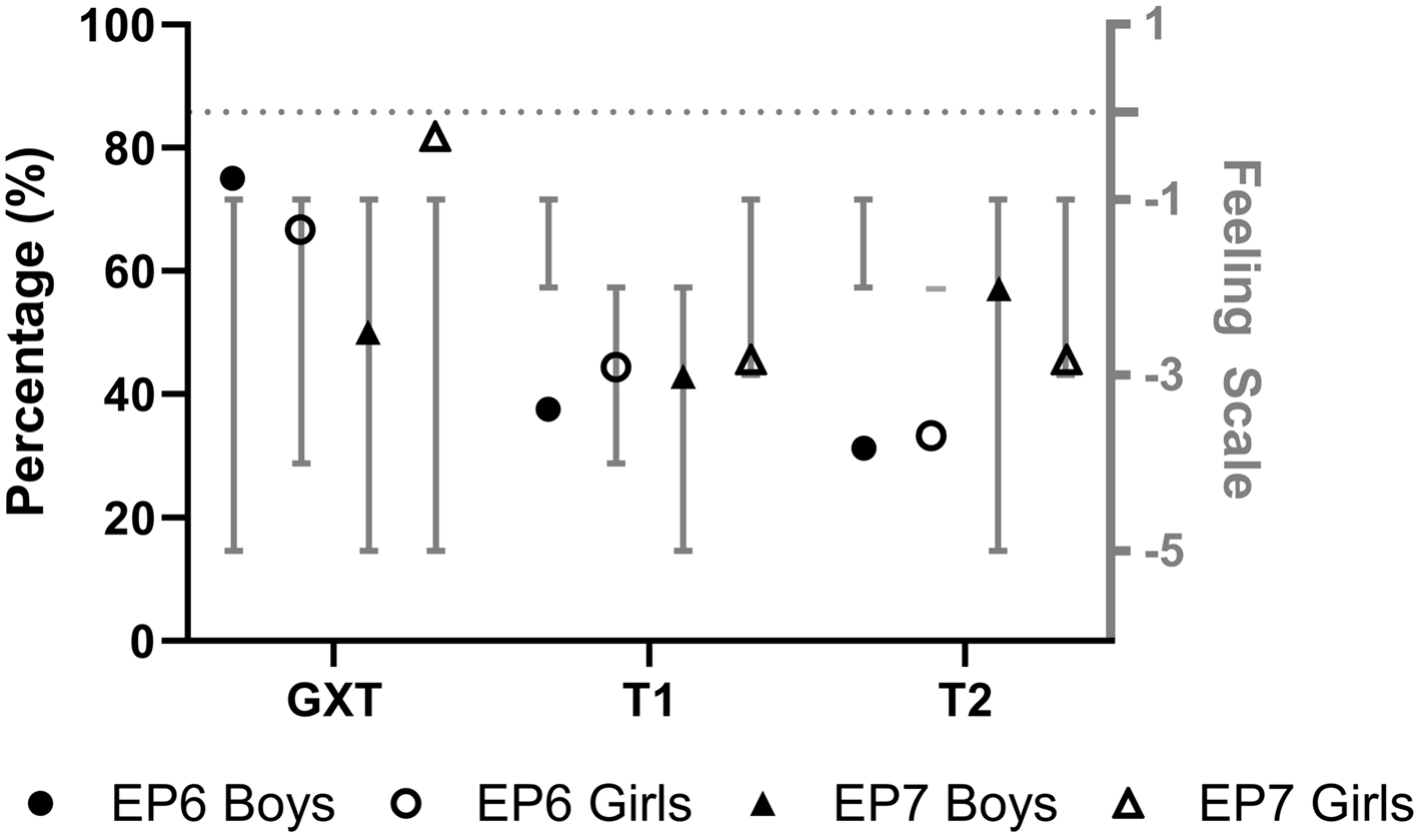
Percentage of participants that reported negative affective responses for the GXT and two submaximal 20mSRT trials stopped at EP6 (*n* = 16 boys, *n* = 9 girls) and EP7* (*n* = 14 boys, *n* = 11 girls). The dotted line depicts neutral affect. The range of reported negative affective responses are shown in the grey bar. Note. * Data obtained from Kasai et al. (2023)

The analysis of interindividual difference in end-test affective responses showed that 72% and 64% of all participants from the EP6 and EP7 groups, respectively, reported negative end-test affect ranging from − 1 to − 5 during the GXT. The submaximal 20mSRTs stopped at EP6 resulted in 40% (range = − 1 to − 4) and 32% (range = − 1 to − 2) of participants reporting negative end-test affective responses during the first and second submaximal 20mSRT trials, respectively. The submaximal 20mSRTs stopped at EP7 found that 44% (range = − 1 to − 5) and 52% (range = − 1 to − 5) of participants reported negative end-test affect in the first and second submaximal 20mSRT trials, respectively (Fig. 2).

## Discussion

The aim of this study was to build on our prior work (Kasai et al. 2023) to explore the validity, test–retest reliability, and affective responses of a submaximal 20mSRT protocol that was stopped at EP6. A secondary aim was to compare data from the two submaximal 20mSRT protocols stopped at EP6 and EP7, with data for the latter obtained from our previously published study (Kasai et al. 2023).

The results of this study showed that a submaximal 20mSRT protocol stopped at EP6 can be used to predict VO_2peak_ accurately. In this study, EP9 provided the most accurate predictions (mean difference = 0.8 mL.kg^−1^.min^−1^) compared to EP10 (mean difference = -1.4 mL.kg^−1^.min^−1^). Interestingly, the mean differences between the measured and predicted VO_2peak_ observed here were very similar to those found in Kasai et al. (2023) where the 20mSRT was stopped at EP7 (mean difference =  ~ − 0.5 to ~ 1.7 mL.kg^−1^.min^−1^). In both studies, EP6 equated to approximately 80% of VO_2peak_, while EP7 was ~ 90% of VO_2peak_. The results of this study therefore show that despite terminating the submaximal 20mSRT at a lower intensity, the accuracy of predictions in VO_2peak_ was not compromised.

The reliability analysis revealed a “moderate” test–retest reliability for the submaximal 20mSRT stopped at EP6. In contrast, the ICC from the submaximal 20mSRTs terminated at EP7 showed “excellent” reliability (Kasai et al. 2023). Therefore, while accurate predictions of VO_2peak_ were obtained from the submaximal 20mSRTs stopped at EP6, the reliability was reduced. However, this reduction in reliability is not surprising. In adults, Faulkner et al. (2007) found higher ICC values when a wider perceptual range was used to predict VO_2peak_. In their study, a perceptual range of RPE9–17 showed the highest test–retest reliability compared to RPE9–13 and RPE9–15 on Borg’s 6–20 RPE scale, which equates to approximately EP1–7, EP1–4, and EP1–6, respectively, on the EP scale.

The results of this study support our hypothesis that the submaximal 20mSRT protocol elicits less aversive end-test affective responses in comparison to a maximal exercise test (i.e. GXT). Affect data showed no significant differences in pre-test affective responses across all three trials. However, the end-test affective responses showed that the submaximal 20mSRT stopped at EP6 was less aversive in comparison to the GXT by a mean difference of ~ 1.5 FS units. This may be clinically significant as even a 1-unit positive shift in affective response during an acute bout of exercise has been shown to predict ~ 30 min of additional MVPA per week in similar aged children (Schneider et al. 2009). Comparison of mean end-test affective responses from both submaximal 20mSRT protocols stopped at EP6 and EP7 (Kasai et al. 2023) showed no significant differences. However, interestingly, there was a significant difference between the two EP6 submaximal 20mSRTs. The relatively small (mean difference =  ~ 0.5 FS units) change in mean end-test affective responses observed in the EP6 protocol may possibly be through improvements in affective associations of the exercise task (Williams and Evans 2014; Brand and Ekkekakis 2018) as observed from the interindividual analysis of affective responses.

The frequency count and the range of affective responses reported demonstrates the high interindividual variability in affective responses during exercise consistent with dual-mode theory (Ekkekakis 2003). During the initial GXT session, 72% of all participants in the EP6 cohort and 64% from the EP7 (Kasai et al. 2023) cohort reported a wide range (range = − 1 to − 5) of end-test negative affective responses. In the EP6 group, during the submaximal 20mSRT sessions, some individuals shifted towards neutral or positive end-test affect. The shift in end-test affect was consistent between both boys and girls in this cohort. Importantly, while ~ 40% of the participants reported negative end-test affect, the range of negativity had diminished by the second 20mSRT trial. In comparison, the EP7 group demonstrated improvements in end-test affective responses in girls from − 1 to − 5 during the GXT to − 1 to − 3 during both the 1st and 2nd submaximal 20mSRT bouts. However, interestingly, the boys from the EP7 cohort showed no improvements in end-test affect during the submaximal 20mSRT sessions. The relatively unchanged negative affective responses in boys from the EP7 group could possibly be explained by a lower preference and/or tolerance to high exercise intensities (Ekkekakis et al. 2005). Schneider and Graham (2009) found that similar aged children who had a preference for lower intensity exercise may report negative affect during exercise of any intensity. As preference and tolerance were not assessed in this study, this explanation is speculative. The results from the exploration of individual end-test affective responses of both submaximal 20mSRT protocols may suggest that the EP6 protocol in this study may be more beneficial in minimising aversive affective responses.

This study has limitations that should be considered when interpreting the results. Estimates of VO_2peak_ were derived using prediction equations based on submaximal RPE-speed relationships. Due to convenience sampling and the nature of the study (i.e. involves maximal exercise), children who prefer exercise could have opted to participate in the study resulting in sampling bias. The test–retest reliability revealed ‘moderate’ to ‘excellent’ reliability based on the ICC estimates alone for this current study and the previously published study (Kasai et al. 2023), respectively. However, it is worth noting that the 95% confidence intervals of the estimates (Koo and Li 2016) may indicate that the true estimate of the ICC may be between ‘poor’ to ‘good’ for the current study and between ‘good’ to ‘excellent’ for the previous study (Kasai et al. 2023). This study was also limited to healthy-weight children and, therefore, the results may not be generalisable to children who may be above a healthy-weight. The cross-sectional comparison of the results from this study and the previously published study (Kasai et al. 2023) were from different time points. Importantly, the 20mSRT is also commonly performed in groups where multiple participants exercise at a given time. According to the Global Explanatory Model for Perceived Exertion (Noble and Robertson 1996), psychosocial/situational mediators (e.g. individuals within the exercise environment) and performance-related mediators (e.g. competitor’s exercise performance) may affect RPE response. Given the results of this study, further research is warranted to explore the ecological validity in group settings, particularly as group dynamics may influence RPE responses (Haile et al. 2015).

The findings from this study may benefit current policies and practice around how the 20mSRT is being utilised in both school and clinical settings. Given the results of this current study and our previously published findings (Kasai et al. 2023), a submaximal 20mSRT utilising RPE to guide test end point can be used to provide a good estimate of VO_2peak_ while being more time efficient and minimising aversive affect without the need for expensive equipment.

Thus, our findings suggest benefits around the use of a submaximal 20mSRT protocol compared with maximal fitness testing. From a practical perspective, given the difference between the two test end points, clinicians, educators, and researchers may need to consider the compromise between test–retest reliability and minimising negative affective responses when deciding which RPE end point to utilise during testing.

## Conclusions

RPE reported during a submaximal 20mSRT protocol stopped at EP6 can be used to predict VO_2peak_ accurately. However, in comparison to the EP7 protocol, test–retest reliability of the EP6 protocol was reduced, although still in the moderate range. The results from this study showed that the EP6 protocol may be more beneficial in avoiding aversive affective responses.

## Data Availability

Data that were presented or mentioned in the manuscript are available from the corresponding author on request.
